# Production and characterization of recombinant pertactin, fimbriae 2 and fimbriae 3 from *Bordetella pertussis*

**DOI:** 10.1186/1471-2180-9-274

**Published:** 2009-12-29

**Authors:** Yinghua Xu, Yaying Wang, Yajun Tan, Huajie Zhang, Lijie Wu, Lichan Wang, Qiming Hou, Shumin Zhang

**Affiliations:** 1 Department of serum, National Institute for the Control of Pharmaceutical and Biological Products, Temple of Heaven, Beijing, 100050, PR China

## Abstract

**Background:**

*Bordetella pertussis *is a causative agent of pertussis or whooping cough in humans. Pertactin (Prn), fimbriae 2 (Fim2) and fimbriae 3 (Fim3) of *B. pertussis *are important virulence factors and immunogens which have been included in some acellular pertussis vaccines. In this present study, we cloned, expressed and purified Prn, Fim2 and Fim3, respectively. The immunogenicity and protective efficacy of the three recombinant proteins (rPrn, rFim2 and rFim3) were investigated in mouse model.

**Results:**

Three recombinant proteins with amount of 12 to 25 mg/L were produced. Compared to the control mice only immunized with adjuvant, serum IgG antibody responses were significantly induced in the mice immunized with rPrn, rFim2 or rFim3 (*P *< 0.001 for all three proteins). Furthermore, T cell responses characteristic of increased production of IL-2 and TNF-α (only for rPrn) were elicited in the mice immunized with the three proteins (*P *< 0.05 for all three proteins). Immunization with rPrn, but not with rFim2 or rFim3, significantly enhanced clearance of bacteria in the lungs of mice after intranasal challenge with *B. pertussis *(*P *< 0.05). When tested in a lethal intracerebral infection model, certain protection was observed in mice immunized with rPrn.

**Conclusions:**

We have developed an efficient method to produce large amounts of rPrn, rFim2, and rFim3 from *B. pertussis*. The three recombinant proteins induced both humoral and cellular immune responses in mice. Immunization with rPrn also conferred protection against pertussis in mouse infection models. Our results indicated that the recombinant proteins still retain their immunological properties and highlighted the potential of the recombinant proteins for the future development of the *B. pertussis *vaccines.

## Background

Pertussis or whooping cough is an infectious respiratory disease caused by the bacterium *Bordetella pertussis*. Despite being preventable by vaccination, pertussis remains one of the top ten causes of death worldwide in childhood, mainly in unvaccinated children [1]. According to the World Health Organization (WHO), about 17.6 million cases of pertussis occurred all over the world and about 279,000 patients died of pertussis in 2003 [2]. Most of deaths occurred in the developing countries.

Immunization with whole cell pertussis vaccines (WPVs) was started in the middle of 20^th ^Century and has significantly reduced the incidence of pertussis in many countries including China [3]. However, these WPVs have drawbacks in causing side effects such as local pain, redness, swelling, fever, and fussiness [4,5]. Due to the fear of side effects and the need for booster immunizations in older age groups, acellular pertussis vaccines (APVs) were developed and they were first introduced in Japan in 1981 [6]. Typically current APVs are comprised of antigens directly purified from cultured *B. pertussis *bacteria. They may include pertussis toxin (PT), filamentous hemagglutin (FHA), Prn, or Fim2 and Fim3. Clinical efficacy trials carried out in Sweden and Italy indicated that APVs containing two or three more components (such as Prn, Fim2 and Fim3) were more effective than the PT alone and/or FHA based vaccines [7,8]. In China, two component APVs containing PT and FHA have been developed and utilized since 1990s [9].

Prn, originally called 69-kDa outer membrane protein, has been shown to play a role in invasion of eukaryotic cells by *B. pertussis *bacteria [10]. It has also reported that Prn elicits both humoral and cellular immune responses in mice and protects infant mice from respiratory challenge by *B. pertussis *[11]. However, the low yield of Prn from cells or the culture supernatant of *B. pertussis *has been a limiting factor in the production of Prn-containing APVs [12]. Fimbriae (Fim), also known as pili and agglutinogen, belong to bacterial adhesins which are expressed on the *B. pertussis *surface. Fim2 and Fim3 are closely related in molecular weight (22 kDa and 22.5 kDa) but are serologically distinct [13-15]. Similar characteristics and molecular weight of Fim2 and Fim3 hampered the production of separate proteins from *B. pertussis *[14,15]. So far there have been no separate purified Fim2 and Fim3 available. In addition, antigenic divergence between vaccine strains and clinical isolates [16-18] as well as the possible presence of other reactogenic contaminants [19], should be considered during purification of those proteins. To overcome these difficulties, attempts have been made to express the proteins *in vitro *by recombinant technology. This technology has advantages regarding of higher yield and controlled production of recombinant proteins at a high homogeneity [20,21]. If such a platform could be established, not only the cost for APV production could be reduced, but also the ability to deal with the antigenic shift could be enhanced.

In this report, we described a method that can be used to produce large amount of rPrn, rFim2 and rFim3 proteins. By using these proteins, we studied their immunogenicity and protective properties in mouse model.

## Results

### Expression and characterization of rPrn, rFim2 and rFim3

**Figure 1:**
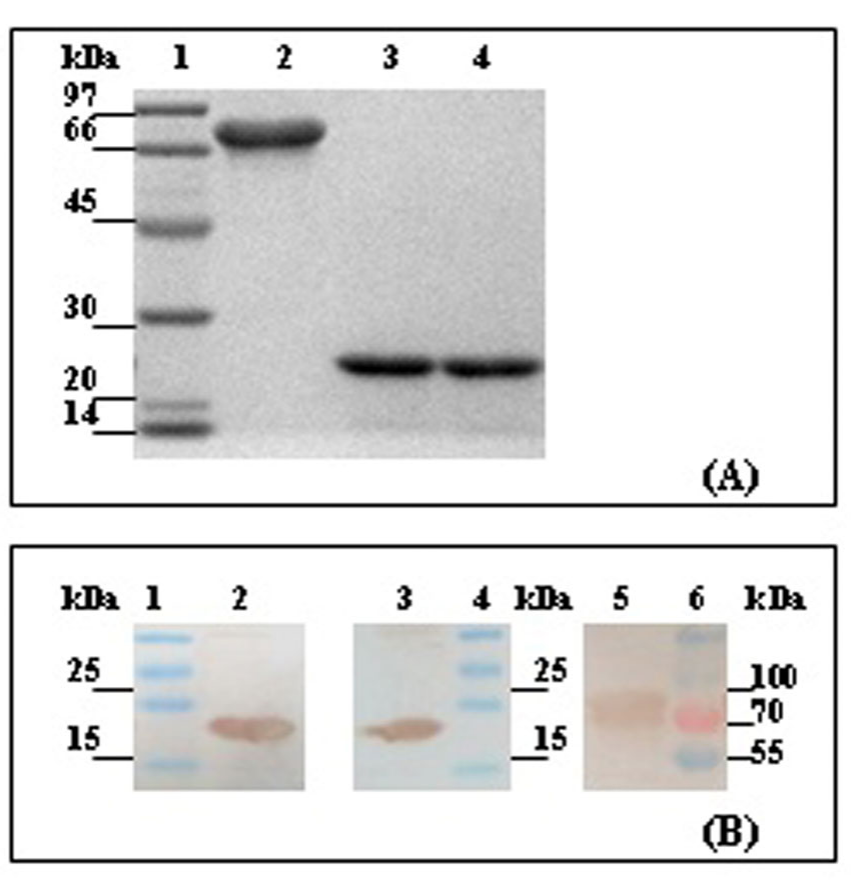
**SDS-PAGE and Western blotting analysis**. **(A) **SDS-PAGE of the purified recombinant proteins. The proteins were electrophoresed on a 10% SDS-PAGE gel under reducing condition and stained by Coomassie blue. Lane 1: Molecular mass marker, the molecular mass standards are indicated in kDa on left side; lane 2: rPrn (10 μg); lane3: rFim2 (10 μg); lane 4: rFim3 (10 μg). **(B) **Western blotting of the recombinant proteins. Lane 1: Pre-stained molecular mass marker (170 kDa, 130, 100, 70, 55, 40, 35, 25, 15, 10, Fermentas), the molecular mass standards are indicated in kDa on left side; lane 2: rFim2 was detected with mouse anti-Fim2 monoclonal antibodies; lane 3: rFim3 was detected with mouse anti-Fim3 monoclonal antibodies; lane 4: Pre-stained molecular mass marker, the molecular mass standards are indicated in kDa on right side; lane 5: rPrn was detected with mouse anti-Prn monoclonal antibodies; lane 6: Pre-stained molecular mass marker, the molecular mass standards are indicated in kDa on right side.

To generate recombinant proteins rPrn, rFim2 and rFim3 in *Escherichia coli*, respective genes were amplified from a Chinese vaccine strain CS and cloned into a protein expression vector. Three expression plasmids were constructed, and the DNA sequences were confirmed by DNA sequencing. Protein expression was induced by isopropyl-β-D-thiogalactopyranoside (IPTG), and purification of the three recombinant proteins was achieved through nickel affinity chromatography with the HisTrapTM HP column. Each purified protein migrated as a single band with the predicted size in SDS-PAGE, of which purity was more than 95% (Figure 1). The specificity of the bands was confirmed by using specific antibodies generated against native forms of Prn, Fim2 or Fim3, respectively, in Western blotting (Figure 1). By using this approach, a large amount of proteins was obtained, at approximately 12 mg/L of rPrn, 25 mg/L of rFim2, and 19 mg/L of rFim3.

### Serum antibody responses to rPrn, rFim2 and rFim3

**Figure 2:**
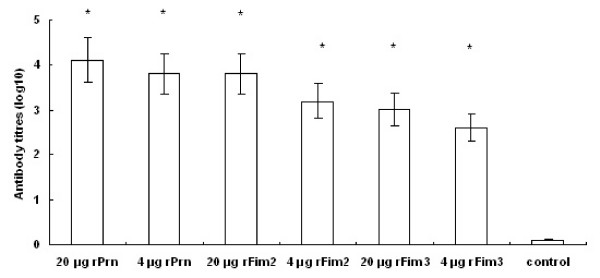
**Antibody responses in immunized and control mice**. Two weeks after the second immunization, sera were collected, and IgG antibody titres were determined by ELISA. Results represent the mean antibody titres for five mice per group. An asterisk symbol (*) indicates a statistically significant difference (*P *< 0.001) between immunized and control group.

In order to examine the antibody responses to rPrn, rFim2 and rFim3, sera of immunized mice were collected two weeks after the second immunization. Titres of serum IgG antibodies were measured by ELISA. Significant IgG antibody responses were observed in the mice immunized with both high and low doses of rPrn, rFim2 or rFim3 when compared to the control group (*P *< 0.001 for all three proteins) (Figure 2). High levels of IgG antibodies were induced in mice immunized with high doses of the three proteins. However, the differences were not significant when compared to those in mice immunized with low doses (Figure 2). When the same amount of rFim2 and rFim3 was used in immunization, IgG responses appeared to be similar between the two groups (*P *= 0.056).

### Serum cytokine responses to the rPrn, rFim2 and rFim3

**Figure 3:**
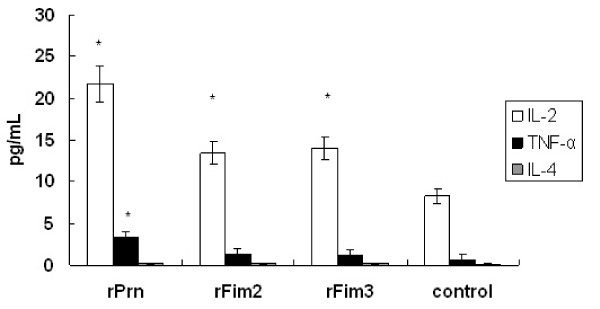
**Cytokine responses in immunized and control mice**. Two weeks after the second immunization, blood samples were collected from five mice from each group. The cytokines were determined by ELISA and are expressed as pg/mL sera. Results are the mean responses for five mice per group. An asterisk symbol (*) indicates a statistically significant difference (*P *< 0.05) between immunized and control group.

Both Th1 (IL-2 and TNF-α) and Th2 (IL-4) cytokine responses were determined after the second immunization with the recombinant proteins. For IL-2, significant higher levels were induced in mice immunized with rPrn, rFim2 or rFim3 when compared to the control mice (*P *< 0.05 for all three proteins). For TNF-α, significant higher level was only observed in mice immunized with rPrn (*P *= 0.037), but not in those with rFim2 or rFim3. The IL-4 induction was not found in all groups of mice (Figure 3).

### Intranasal challenge with *B. pertussis*

**Figure 4:**
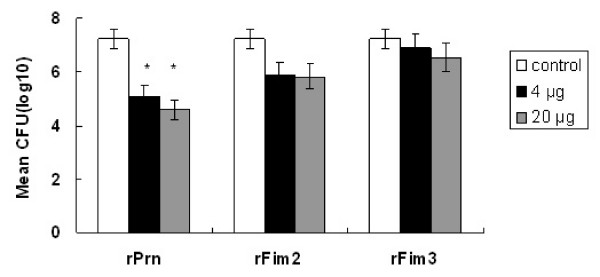
**Protection against intranasal challenge with *B. pertussis***. Two weeks after the second immunization, the mice were challenged intranasally with *B. pertussis *18323, and CFU counts were performed on individual lung homogenate. Results are mean viable *B. pertussis *counts from five mice per group. An asterisk symbol (*) indicates a statistically significant difference (*P *< 0.05) between immunized and control group.

Seven days after the intranasal challenge with *B. pertussis*, the bacterial loads were significantly lower in the lungs of mice immunized with high or low doses of rPrn, compared to those observed in the control mice (*P *= 0.021 and *P *= 0.039). For the mice immunized with rFim2 or rFim3, no significant difference was observed in the bacterial loads in the lungs compared to the control mice (Figure 4).

### Intracerebral challenge with *B. pertussis*

**Figure 5:**
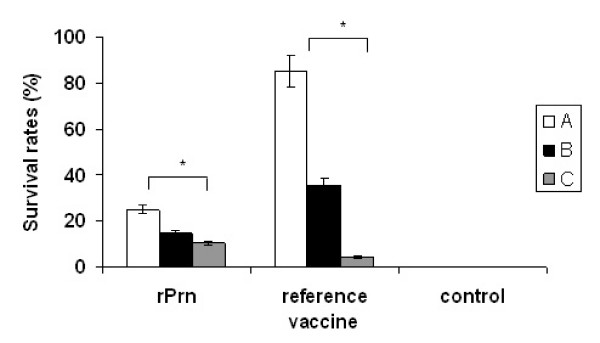
**Protection against intracerebral challenge with *B. pertussis***. Three weeks after immunization, all mice (sixteen mice per group) were challenged intracerebrally with a lethal dose of *B. pertussis *18323, and survival of challenge mice was monitored. For recombinant proteins immunized groups, A, B, and C indicated 100 μg, 20 μg, and 4 μg dose of immunization. The reference vaccine is used as national reference standard in the intracerebral challenge assay in China and this standard have an assigned activity of 14 IU/ampoule. A, B and C indicated 0.5 IU, 0.1 IU and 0.02 IU dose of immunization. All mice of control group were immunized adjuvant alone. An asterisk symbol (*) indicates a significant difference (*P *< 0.05) between immunized and control group.

Two weeks after the intracerebral challenge with a lethal dose of *B. pertussis*, none of the mice in the control group survived (Figure 5). In contrast, a dose-dependent protection was observed in mice immunized with different doses of the reference vaccine. For the mice immunized with rPrn, some protection against the lethal dose of intercerebral challenge was noticed when compared to the control mice (*P *= 0.005). The level of this protection provided from immunization with rPrn was clearly higher than that from the immunization with 0.02 IU of reference vaccine (*P *= 0.027). The result suggested that immunization with rPrn alone can confer partial protection against a lethal intracerebral *B. pertussis *challenge. Such intracerebral challenge assays were also performed in the groups immunized with different doses of rFim2 and rFim3. However, no significant protection was observed as none of mice were survived in the groups immunized with 20 μg dose of rFim2 and 4 μg dose of rFim3 and a few (less than three) survival mice in other dose groups.

## Discussion

Because of its advantages in cost, yield and purity, vaccine based on recombinant components has been considered to be a valuable alternative for the vaccine production [22], in particular for the developing countries. In the present study, we showed that the recombinant Prn, Fim2 and Fim3 proteins can be readily expressed and purified in large quantities from *E. coli*, and each recombinant protein solution is stable for up to twelve months when stored at below -20°C. They were prepared in a large quantity and freeze-dried. It was confirmed that the activity of freeze-dried preparation had no difference significantly compared with liquid preparation by ELISA method and in some animal experiments (data not shown). The three recombinant proteins can elicit both humoral and cellular immune responses when they were investigated in mice. Furthermore, this recombinant technology makes it possible to avoid contaminations from the *B. pertussis *components that may cause side effects in vaccine preparations [19]. Availability of the purified Fim2 and Fim3 also provided an opportunity to assess their individual roles in the immunogenicity and protective efficacy.

As a virulence factor of *B. pertussis*, the ability of Prn to function as adhesin has been investigated both *in vitro *and *in vivo *[10,23]. It is reported that the Prn-mediated protection may be afforded by blocking Prn-mediated attachment of *B. pertussis *to the host cells [24,25]. Studies on the immunized children have also suggested that high level of circulating antibodies against Prn are associated with protection [26,27]. Furthermore, evidence suggests that anti-Prn antibodies may promote extracellular killing with complement or as opsonins, and mediate killing bacteria by phagocytes [25]. However, although antibodies specific to *B. pertussis *antigens confer protection, many studies have indicated that humoral immunity alone is not sufficient to provide long-term protection against *B. pertussis *infection and that the protection against *B. pertussis *requires both T cell- and B cell-mediated immunity [28,29]. Our results showed that the antibody response increased significantly in mice immunized with rPrn. Immunization of rPrn also induced a Th1 response that is characterized by the enhanced production of IL-2- and TNF-α. These results also indicated that the rPrn shared a similar feature with the native form of Prn, suggesting that B cell and T cell epitopes might be highly conserved in rPrn.

Murine intranasal and intracerebral challenge assays have been validated and used to demonstrate the protection of pertussis vaccine for many years [30-32]. The results obtained from the intranasal and intracerebral challenge tests strongly suggest that rPrn functions as a protective antigen. These observations are consistent with previous reports that a higher Th1-type response was associated with a stronger level of protection against *B. pertussis *[29]. In this study, the bacterial loads were only evaluated on day 7 in lungs of the mince after the intranasal challenge. However, a time course of infection would probably provide more information on the protective properties of the proteins studied.

So far, twelve, two and four different variants have been reported in Prn, Fim2 and Fim3, respectively [17,18,33]. At present, the prevalent allele combinations of *B. pertussis *isolates are *prn2*/*fim3B *[18]. The strains used in this study and the strains used for vaccine production are *prn1*/*fim3A *or *prn6*/*fim3A*. As the difference occurred between *B. pertussis *vaccine strains and circulating isolates in many countries [16-18,33], it has been proposed that the strain variation may have effect on the vaccine efficacy [16]. In this case, engineering strategies will remedy antigenic shifts by performing genetic mutation on the antigen encoding genes, which is an advantage of using recombinant proteins compared with the ones purified from *B. pertussis*.

Because of the similarity in the molecular weight, it is extremely difficult to purify separately Fim2 and Fim3 proteins from *B. pertussis*. Therefore, antibody responses against Fim2 or Fim3 were only measured in ELISA using a mixture of Fim2 and Fim3 proteins as coating antigen in clinical vaccine trials [8,34]. The exact role of Fim2 and Fim3 in protection against pertussis is not fully known. In this study, recombinant Fim2 and Fim3 were expressed and purified separately. For the first times, their functions in protection against pertussis were assessed separately in mice model. The study demonstrated that higher antibody titres and cellular immune response characteristic of increased production of IL-2 were induced in mice immunized with rFim2 and rFim3. Although monoclonal anti-Fim2 and anti-Fim3 antibodies were used in the study, it remains to be shown whether there is cross-reacting response between Fim2 and Fim3.

It is known that IL-2, TNF-α and IFN-γ are characteristic cytokines for Th1 response, and IL-4 and IL-10 for Th2 response [29]. In this study, we have only measured serum concentrations of IL-2, TNF-α and IL-4. It is interesting to study concentrations of other cytokines such as IFN-γ in sera collected from mice after immunization and infection. Further, serum IL-4 was not measurable in all mice tested in this study. This was in contrast to the finding that IL-2 and TNF-α were significantly induced. The exact reason for the undetectable IL-4 was unknown. One explanation might be the NIH mice used in this study. It is known that NIH mice predominate on cellular immunity. Another explanation might be timing of the serum sampling and possible posttranscriptional regulation of IL-4. No matter if IL-4 was measurable or not, anti-pertussis antibodies were significantly induced in mice immunized with each of the three recombinant proteins.

Previous vaccine efficacy trial in Sweden indicated that inclusion of Prn, Fim2 and Fim3 into acellular vaccine containing PT and FHA provided higher protection against pertussis. However, the contribution of individual components in the protection was not revealed [8]. Since Fim of *B. pertussis *facilitates a variety of binding capabilities as adhesins [35], some studies suggested that passive protection against *B. pertussis *infection might be conferred due to the existence of higher titres of anti-Fim2 or anti-Fim3 antibodies which might transmigrate into the lower respiratory tract in mice [36,37]. In contrast, the results from intranasal and intracerebral challenges with *B. pertussis *indicated very limited role played by rFims in bacterial clearance, although higher titres of anti-Fim antibodies have been observed in this study. These data suggest that rFim2 or rFim3 alone may not be enough to provide the protection against *B. pertussis *and that they should be used in combination with other vaccine components such as PT, FHA, and/or Prn.

## Conclusions

*B. pertussis *proteins Prn, Fim2, and Fim3 can be genetically manipulated and expressed in a large amount *in vitro*. The three recombinant proteins can elicit both humoral and cellular immune responses. Immunization with rPrn can confer certain protection in mouse infection models. These recombinant proteins, especially rPrn, have a potential for the development of a new generation of APVs in developing countries such as China.

## Methods

### Bacterial strains and culture conditions

*B. pertussis *strain CS (*prn/fim2/fim3 *allele type: 1/1/A), a Chinese strain isolated in Beijing and used for production of pertussis vaccine, has been described previously [9]. Genomic DNA of this strain was used to generate recombinant proteins. *B. pertussis *strain 18323 (*prn/fim2/fim3 *allele type: 6/1/A), an international reference strain, was used in the mouse intranasal and intracerebral challenge assays. *B. pertussis *strains were grown at 37°C on Bordet-Gengou (BG) agar (Difco) medium supplemented with 20% defibrinated sheep blood. *E. coli *strains BL21 (DE3) (Novagen, Germany) and M15 (Qiagen, Germany) were used for the protein expressions. They were cultured in Luria Broth (LB) medium at 37°C.

### Recombinant protein expression and purification

Construction of recombinant DNA fragments, protein expression and purification were performed as described previously [38]. Briefly, DNAs encoding Prn and Fim3 were amplified by PCR with primers listed in Table 1 from genomic DNA of *B. pertussis *strain CS and ligated into pQE30 vector (Qiagen, Germany) with restriction sites *Bam*HI and *Hind*III. The generated plasmids were designated pQE30/Prn and pQE30/Fim3. By using a similar approach, DNA encoding Fim2 was amplified by PCR and ligated into pET30a (+) (Novagen, Germany) with *Nde*I and *Xho*I restriction sites. The plasmid was named as pET30a (+)/Fim2. The three constructed plasmids were transformed into *E. coli *BL21 (DE3) or M15, respectively. The cloned DNA sequences were verified by DNA sequencing analysis. The nucleotide sequences of *fim2 *and *fim3 *have been submitted to GenBank with accession numbers AY845256 and AY845257.

**Table 1:** Primers used in the study

Gene	Size (bp)	Primer	Sequences (5'-3')
Prn	2031	Prn-p1	CATAGGATCCGACTGGAACAACCAGTCCATCGTCA
		Prn-p2	CAGAAAGCTTGCCGCCGTCGCCGGTGAAGCCG
Fim2	543	Fim2-p3	CATACATATGGACGACGGCACCATCGTCATCACCGGC
		Fim2-p4	GTAACTCGAGGGGGTAGACCACGGAAAAACCCACATA
Fim3	546	Fim3-p5	CTATGGATCCGCGCTGGCCAACGACGGCACCATCGTC
		Fim3-p6	ACTTAAGCTTGGGGTAGACGACGGAAAAGCCGACGTA

The restriction site is underlined

Expression of the recombinant proteins was induced by addition of IPTG to a final concentration of 1 mM. Expressed proteins were purified using the HisTrap™ HP column by the AKTA system (Amersham Pharmacia, USA) according to the manufacturer's recommendations. Briefly, the cells expressing recombinant proteins were collected by centrifugation, and the pellets were sonicated on ice-bath. The inclusion bodies of the recombinant proteins were separated by centrifugation at 12,000 × *g *for 10 minutes at 4°C and solubilized in a buffer solution (pH = 7.4) containing 10 mM Na_2_HPO_4_, 10 mM NaH_2_PO_4_, 500 mM NaCl and 8 M urea. Protein renature was processed by gradually decreasing the concentration of urea to 0.5 M with dialyzing for 48 hours. The proteins were then purified by passing through a Ni2^+ ^affinity chromatography. A binding buffer (10 mM Na_2_HPO_4_, 10 mM NaH_2_PO_4_, 500 mM NaCl, 20 mM imidazole, 0.5 M urea, pH 7.4) and an elution buffer (10 mM Na_2_HPO_4_, 10 mM NaH_2_PO_4_, 500 mM NaCl, 200 mM imidazole, 0.5 M urea, pH 7.4) were used for the protein binding and elution procedures. The purity of each recombinant protein was estimated by 10% SDS-PAGE and densitometry analysis, while the protein concentration was determined by the Lowry method as described previously [38].

### Western immunoblotting

Western immunoblotting was performed as described by Towbin *et al *[39]. In brief, recombinant proteins were separated by SDS-PAGE and transferred onto nitrocellulose membranes using a semi-dry western transfer apparatus (Bio-Rad, USA) at a constant voltage (20 V). Non-specific binding sites of the membranes were blocked by incubation with 5% skim milk (Fluka, USA) in phosphate-buffered solution (PBS) (pH 7.4) containing 0.05% Tween 20 for 1 h. The blots were then incubated with the specific anti-Prn, anti-Fim2 or anti-Fim3 antibodies, kindly provided by Dr. Dorothy Xing, National Institute for Biological Standards and Control, UK. The blot signals were captured by using a DAB kit (Boster, China) following incubation with horseradish peroxidase-conjugated anti-mouse secondary IgG (Jackson, USA).

### Immunization of mice

Male and female NIH mice, at 17-20 days old of age, were obtained from the animal center at the National Institute for the Control of Pharmaceutical and Biological Products (Beijing, China). For the immunogenic study and intranasal challenge assays, mice were divided into seven groups (ten female mice in each group). Each mouse was immunized intraperitoneally on day 0 and 14 with 0.5 mL of each recombinant protein at two concentrations (20 or 4 μg/mouse), absorbed with adjuvant Al(OH)_3 _(0.5 mg per mouse). In control group, ten mice were only immunized intraperitoneally with Al(OH)_3 _(0.5 mg per mouse). Two weeks after the second immunization (day 28), five mice from each group were challenged intranasally, and serum samples were collected from the remaining five mice.

For the intracerebral challenge assays, thirteen groups of mice, consisting eight male and eight female mice in each group, were used. Each mouse was immunized intraperitoneally with 0.5 mL of either different concentrations (100, 20, or 4 μg/mouse) of each recombinant protein formulated with adjuvant Al(OH)_3 _(0.5 mg per mouse), or with a reference vaccine at different doses (0.5, 0.1, or 0.02 IU/mouse). The reference vaccine is a lyophilized WPV which is being used as a national standard of the intracerebral challenge assay for the potency test of APVs in China [40]. The vaccine has an assigned activity of 14 IU/ampoule. Sixteen NIH mice (female and male in half) that were only immunized intraperitoneally with Al(OH)_3 _alone were used as a control group.

The experiments were supervised by the Animal Ethic Committee of National Institute for the Control of Pharmaceutical and Biological Products, Beijing.

### Antibody measurement

Mouse serum antibodies against rPrn, rFim2 and rFim3 were measured by enzyme-linked immunosorbent assays (ELISA). Microtiter plates (Greiner, Germany) were coated with 50 μL of 0.05 M carbonate buffer (pH 9.6) containing 5 μg/mL of the purified recombinant protein. After blocking with PBS containing 0.05% Tween 20 and 1% bovine serum albumin, 50 μL of anti-serum was added in two-fold serial dilutions. Following incubation for 1 h at 37°C, goat anti-mouse IgG conjugated with horseradish peroxidase (Pierce, USA) were added to the plates. After another incubation at the same condition, signals were measured by using 2, 2'-azinobis (3-ethylbenzthiazolinesulfonic acid) (ABTS, Boehringer Mannheim, Germany) substrate according to the manufacturer's instruction. Results were expressed as the highest dilution yielding the absorbance at 405 nm three times above the control values.

### Cytokine measurement

Cytokine concentrations of IL-2, IL-4 and TNF-α in sera of the mice immunized with 20 μg dose of recombinant antigens were determined using a sandwich ELISA kit (Boster, China) according to the manufacturer's instruction. The detection limit of these three cytokines was 2.4 pg/ml for IL-2, 3.1 pg/ml for IL-4 and 1.6 pg/ml for TNF-α, respectively.

### Intranasal challenge with *B. pertussis*

Two week after the second immunization, five mice from each group were challenged intranasally with the *B. pertussis *strain 18323 according to the method described by Cheung *et al *[30]. Bacteria were subcultured on BG agar medium containing 20% defibrinated sheep blood and resuspended in PBS with 1% casamino acids. For each anesthetized mouse, 50 μL of bacterial suspension at approximately 1 × 10^6 ^CFU was injected into the nostril. On day 7 after the injection, lungs of each mouse were removed and homogenized in 1 mL of PBS. Bacterial viable counting was performed by plating serial dilutions on BG agar medium.

### Intracerebral challenge with *B. pertussis*

The *B. pertussis *strain 18323 was used for the intracerebral challenge assay for the immunized mice. Bacterial suspension (30 μL) with approximately 8 × 10^4 ^CFU suspended in PBS containing 1% casamino acids were injected into the mouse brain using a 0.25-mL glass syringe. Animal survival number was enumerated at 14 days post challenge.

### Statistical analysis

All analyses were performed by use of SPSS 13.0 software. One-way analysis of variance followed by Dunnett's (two-sided) test was utilized for the statistical analysis of the host immune responses and protection against intranasal challenges with *B. pertussis *in mice. To compare the difference for the protective efficacies against intracerebral challenge with a lethal dose of *B. pertussis*, the data were analyzed by a Chi-Square test (Mantel-Haenszed Method). *P*-value < 0.05 was considered statistically significant.

## Abbreviations

Prn: pertactin; Fim2: fimbriae 2; Fim3: fimbriae 3; WPVs: whole cell pertussis vaccines; APVs: acelluar pertussis vaccines; CFU: colony forming unit; IPTG: isopropyl-β-D-thiogalactopyranoside.

## Competing interests

The authors declare that they have no competing interests.

## Authors' contributions

SZ and YX conceived the study. SZ, YX, and YW designed the experiments. YX, YW, LJW, LW and QH performed the molecular biological work and the animal studies. YT and HZ performed the statistical analyses and prepared the figures. YX and YW wrote the draft of the manuscript. SZ, YT, and HZ revised the manuscript. All authors read and approved the final version of the manuscript.
